# Early-life maturation of the somatosensory cortex: sensory experience and beyond

**DOI:** 10.3389/fncir.2024.1430783

**Published:** 2024-07-08

**Authors:** Ijeoma Nwabudike, Alicia Che

**Affiliations:** Department of Psychiatry, Yale University School of Medicine, New Haven, CT, United States

**Keywords:** somatosensory perception, barrel cortex development, early life experience, sensory deprivation, REM Sleep, early social isolation, cross-modal sensory experience

## Abstract

Early life experiences shape physical and behavioral outcomes throughout lifetime. Sensory circuits are especially susceptible to environmental and physiological changes during development. However, the impact of different types of early life experience are often evaluated in isolation. In this mini review, we discuss the specific effects of postnatal sensory experience, sleep, social isolation, and substance exposure on barrel cortex development. Considering these concurrent factors will improve understanding of the etiology of atypical sensory perception in many neuropsychiatric and neurodevelopmental disorders.

## 1 Introduction

Early life experiences can have profound and life-long physical, emotional, and behavioral consequences (Felitti et al., 1998; Hughes et al., 2017; Lokhandwala and Spencer, 2022). During postnatal development, maturation of circuits involved in sensory perception occurs first, and this process is particularly sensitive to physiological and environmental influences (Chang and Merzenich, 2003; Maitre et al., 2017; Badde et al., 2020; reviews: Hensch, 2004; Fox et al., 2010). In rodents, whisker-mediated tactile input is one of the earliest developed sensory modalities (Akhmetshina et al., 2016; Smirnov and Sitnikova, 2019). Tactile inputs are present at birth, while visual and auditory inputs do not occur until the second postnatal week in mice (Colonnese et al., 2010; Akhmetshina et al., 2016; Makarov et al., 2021). This feature allows tactile sensory inputs to interact with intrinsic programs to uniquely shape sensorimotor circuit maturation (Bragg-Gonzalo et al., 2021).

The developing somatosensory cortex is dominated by synchronous neural activity until the end of the second postnatal week (Khazipov et al., 2004; Minlebaev et al., 2007; Golshani et al., 2009; Tolner et al., 2012; Yang et al., 2013). Its precise patterns and timing are essential for many activity-dependent processes during early postnatal development (Winnubst et al., 2015; Wong et al., 2018; Duan et al., 2020; review: Leighton and Lohmann, 2016). Such activity has been suggested to originate from at least four sources: (1) external, passive tactile sensory inputs onto the whiskers from the dam, littermates, and nesting materials (Akhmetshina et al., 2016); (2) sensorimotor feedback generated by involuntary whisker and limb twitches (Khazipov et al., 2004; Tiriac et al., 2014; Dooley et al., 2020); (3) cross-modal sensory inputs, such as odor-driven activity via direct early excitatory connections from the olfactory cortex to somatosensory cortex (Henschke et al., 2018; Cai et al., 2024); 4) internally generated, spontaneous neural activity independent from the periphery or movement (Moreno-Juan et al., 2017; Mizuno et al., 2018; Nakazawa et al., 2020; Banerjee et al., 2022). Multiple lines of evidence support that activity from these different sources co-exist in the first two postnatal weeks. While spontaneously occurring and whisker stimulation-induced oscillatory activity are both present already at birth, inactivation of the tactile sensory periphery with lidocaine, which occludes tactile sensory inputs and sensorimotor feedback from whisker twitches, reduces but does not abolish synchronous activity (Yang et al., 2009). In addition, barrel column-related patchwork activity in L4 excitatory neurons is uncorrelated with whisker movement and does not disappear after infraorbital nerve transection at P4 (Mizuno et al., 2018; Nakazawa et al., 2018). There is still considerable ongoing debate over whether the four sources of activity mentioned above are distinct – for example, what proportion of activity that has been attributed as “internally-generated activity” is due to sensorimotor feedback given that the circuitry enabling movement-related sensory feedback is already functional in the thalamus embryonically (Antón-Bolaños et al., 2019; Dooley et al., 2020; Blumberg et al., 2022). Latest data demonstrating that synchronous activity in the barrel cortex can be driven by chemosensation also raises the possibility that the remaining synchronous activity after inactivation of tactile sensory periphery could have, at least in part, originated from other sensory modalities (Cai et al., 2024; Wang et al., 2024). Further investigations will be required to confirm or resolve potential overlaps between sources of early neural activity in the barrel cortex.

**FIGURE 1:**
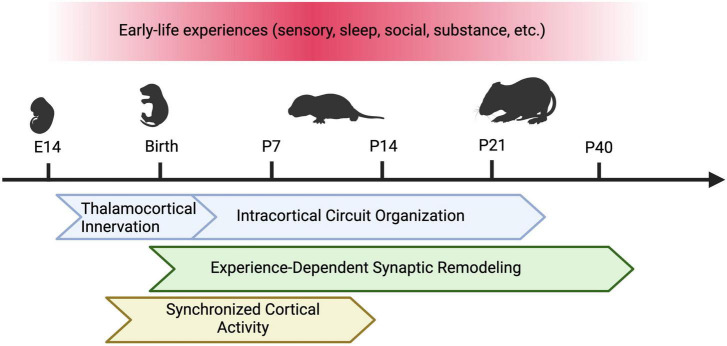
Types of Early Life Experiences and Susceptible Developmental Programs in the Barrel Cortex. In rodents, early sensory experience, sleep, social isolation, and perinatal substance exposure can alter the development of the barrel cortex. Different developmental programs may be affected by these factors across varying sensitive periods. These programs include thalamocortical innervation, intracortical circuit assembly (including maturation of excitatory and inhibitory circuits), experience-dependent synaptic remodeling, and synchronous cortical activity. Figure created with Biorender.com.

Given the complexity of early neural activity as discussed above, in this minireview we will focus on summarizing the role of the following early-life sensory experiences on the development of somatosensory cortex: (1) external tactile sensory inputs; (2) sleep in the context of permitting sensorimotor feedback; (3) recently discovered cross-modal olfactory sensory inputs. While there might be overlap in mechanisms, we will not focus on internally generated intrinsic activity here (review: Leighton and Lohmann, 2016). We will also discuss recent findings that have uncovered the impact of other early-life factors, including isolation and substance exposure (Figure 1).

## 2 Neonatal tactile sensory experience

In the whisker sensory system, tactile sensory input to the whiskers is transduced by mechanoreceptors in each whisker follicle on the snout, then carried by afferent fibers to the trigeminal nucleus of the brainstem, the ventroposteromedial nucleus of the thalamus (VPM) and finally to the somatosensory cortex (reviews: Petersen, 2007; Diamond et al., 2008). In layer 4 (L4) of the barrel cortex, cytoarchitectonic cylindrical structures known as “barrels” correspond to individual whiskers (Woolsey and van der Loos, 1970). Synaptic connections of within barrel cortex have been extensively characterized, making it an excellent model for investigating experience-dependent circuit maturation. Peripheral manipulations have been widely used and demonstrated to produce marked alterations in the structure and function of the developing barrel cortex. These studies provide a wealth of information on the age-, layer-, cell type-, and synaptic connection-specific effects of postnatal sensory experience (review: Erzurumlu and Gaspar, 2012). Below we will summarize effects of tactile sensory deprivation via peripheral manipulations on barrel cortex synaptic connectivity (thalamocortical and intracortical), neuronal maturation, and behavior.

Thalamocortical afferents (TCAs) to the barrel cortex play a critical role in instructing barrel formation and organization, dendritic remodeling, and neuronal maturation (Li et al., 2013; Matsui et al., 2013; Pouchelon et al., 2014; Young et al., 2023). Disruption of presynaptic vesicle fusion, glutamatergic neurotransmission, or cAMP signaling at thalamocortical synapses alters barrel patterning (Abdel-Majid et al., 1998; Iwasato et al., 2000; Inan et al., 2006; Ballester-Rosado et al., 2010; Narboux-Nême et al., 2012; Li et al., 2013; Suzuki et al., 2015). The TCAs are also particularly sensitive to sensory deprivation. When sensory input is eliminated by infra-orbital nerve transection or whisker follicle cauterization before postnatal day (P) 3, TCA arbors no longer cluster and cylindrical barrels are replaced by elongated fused bands that extend through L4 of the barrel field (Van der Loos and Woolsey, 1973; Killackey and Belford, 1979; Wong-Riley and Welt, 1980; Jeanmonod et al., 1981; Jensen and Killackey, 1987). Similar manipulations that result in permanent peripheral damage at later timepoints, between P4 and P6, lead to TCA disorganization but not a complete loss of barrel structure, while normal barrel patterns are preserved when manipulations occur after P6 (Woolsey and Wann, 1976; Belford and Killackey, 1980; Jeanmonod et al., 1981; Bates and Killackey, 1985; Higashi et al., 1999). Neonatal whisker trimming or plucking, which limits tactile sensory experience without causing peripheral structural damage, does not alter the cytoarchitectonic barrel structure; instead, it leads to changes in the strength of thalamocortical and intracortical synapses (Finnerty et al., 1999; Allen et al., 2003; Sadaka et al., 2003; Schierloh et al., 2004; Bender et al., 2006; Chittajallu and Isaac, 2010; Simons et al., 2015). For instance, the density and efficacy of thalamocortical synapses decrease following chronic whisker trimming (Sadaka et al., 2003). Following extended whisker regrowth, thalamic drive to excitatory neurons rebounds to above control levels, suggesting that sensory inputs can restore and even heighten the efficacy of thalamocortical synapses after early-life manipulations (Simons et al., 2015). Sensory deprivation also weakens intracortical synapses at L4-to-L2/3 connections and increases lateral synaptic transmission across barrel columns, resulting in increased neuronal responses to deflection of surrounding non-principal whiskers (Fox, 1992; Finnerty et al., 1999; Lendvai et al., 2000; Stern et al., 2001; Allen et al., 2003; Shepherd et al., 2003; Schierloh et al., 2004; Bender et al., 2006; Lee et al., 2007). When whisker regrowth is allowed, L4 neurons continue to show broadened sensory-evoked responses (Simons and Land, 1987; Shoykhet et al., 2005). In summary, these studies establish that sensory experience from the periphery plays a central role in regulating thalamocortical and intracortical circuit assembly during development.

During the early postnatal period, cortical GABAergic interneurons also receive significant thalamic inputs and shape circuit function in a sensory experience-dependent manner. In the first postnatal week, direct TCAs from the VPM preferentially form synaptic connections with select interneuron subtypes, such as somatostatin-expressing (SST) interneurons in L5 and Reelin-expressing (Re) interneurons in L1 (Marques-Smith et al., 2016; Tuncdemir et al., 2016; Che et al., 2018; Pouchelon et al., 2021). These thalamic inputs to interneurons are dynamically remodeled during development−VPM inputs to SST, Re, and Vasoactive intestinal peptide-expressing (VIP) interneurons weaken by the end of the second postnatal week, while VPM inputs to parvalbumin-expressing (PV) interneurons and PV-driven feed-forward inhibition continue to strengthen (Daw et al., 2007; Che et al., 2018; Kastli et al., 2020; Modol et al., 2020). The transient TCA connections are required for the maturation of stable circuit connectivity that persists into adulthood (Anastasiades et al., 2016; Marques-Smith et al., 2016; Tuncdemir et al., 2016). It has been hypothesized that interneurons are more sensitive to early-life sensory experience due to their protracted postnatal development (review: Micheva and Beaulieu, 1997). This hypothesis is supported by recent studies demonstrating that sensory deprivation specifically reduces VPM to interneuron inputs and interneuron activity, resulting in delayed cell maturation and alterations in intracortical connectivity (Chittajallu and Isaac, 2010; Marques-Smith et al., 2016; Che et al., 2018; Duan et al., 2020; Bollmann et al., 2023). Whisker plucking from the first postnatal week to adulthood decreases L4 surround inhibition, inhibitory synaptic strength, and the total number of interneurons (Micheva and Beaulieu, 1995a,b; Shoykhet et al., 2005; Gainey et al., 2016). Taken together, these studies demonstrate that developing interneurons are uniquely positioned to relay thalamic inputs and regulate responses to early sensory experiences, thus playing a significant role in shaping cortical circuit formation and function.

Early sensory deprivation also has profound behavioral consequences. Infant-trimmed rodents can differentiate between rough and smooth textures but show difficulty distinguishing between two distinct rough textures (Carvell and Simons, 1996). This is thought to be a consequence of permanent deficits in surround inhibition, which decreases the animal’s perceptual ability in tasks that require information from multiple whiskers (Carvell and Simons, 1996; Shoykhet et al., 2005). Trimming also alters explorative whisking and behavioral strategies during gap-crossing tasks (Lee et al., 2009; Papaioannou et al., 2013). Intriguingly, the effects of early sensory deprivation are especially evident in socio-cognitive behavioral tasks. Animals that have been vibrissectomized are more social and explorative, displaying less emotional reactivity and diminished early withdrawal response from novel tactile stimuli (Shishelova, 2006; Lee et al., 2009; Soumiya et al., 2016; Wang et al., 2022). Furthermore, sensory deprivation through whisker removal reduces excitatory synaptic transmission as well as the synthesis and secretion of the neuropeptide oxytocin, while both oxytocin injection and increased sensory experience rescues excitatory synaptic transmission (Zheng et al., 2014). Nevertheless, it remains to be determined through what specific mechanisms early tactile sensory experience influences the development of higher-order social, emotional, and cognitive function.

## 3 Sleep and sleep deprivation

Similar to human infants, neonatal rodents spend the majority of their time sleeping (Blumberg et al., 2022). The younger the animal, the more time it spends in active (rapid-eye-movement, REM) sleep states (Jouvet-Mounier et al., 1969). During the first two weeks of life, rodents spend between 45 to 80% of their time in REM sleep, 20 to 35% in wakeful states, and up to 25% in non-REM sleep, while in adult rodents, REM sleep time decreases to 8.5% and time spent awake rises to 43% (Jouvet-Mounier et al., 1969; Blumberg et al., 2022). As discussed previously, sensorimotor feedback generated by myoclonic twitches is a main source of early synchronous activity in the barrel cortex (Blumberg et al., 2022). Recent studies correlating electrophysiological recordings of barrel cortex activity with whisker motion show that up to 75% of spontaneous somatosensory cortical activity is directly related to passive whisker movement (Akhmetshina et al., 2016; Dooley et al., 2020). These twitches during REM sleep are more likely to generate network activity in the barrel cortex than movements during wakefulness (Dooley et al., 2020). Therefore, REM sleep during development serves to enable muscle twitches important for activity-dependent circuit maturation in the barrel cortex. In support of this, REM sleep during development has been shown to support heightened synaptic remodeling, in particular synaptic elimination (Yang and Gan, 2012; Li et al., 2017; Zhou et al., 2020; review: Sun et al., 2020), a process essential for the development of mature and functional circuits (Faust et al., 2021). In addition, REM sleep increases coherence between distant brain regions, facilitating the formation of long-range connections that direct complex sensory-based behaviors in adulthood (Rio-Bermudez et al., 2020).

Early-life sleep deprivation may also alter inhibitory circuits – persistent decrease in PV interneuron number has been reported in adult voles whose sleep was disrupted between P14 and P21 (Jones et al., 2019). Behaviorally, prairie voles that were sleep-deprived as neonates display aberrant exploratory behavior and pair bond formation during adulthood (Jones et al., 2019). Together, these findings indicate that REM sleep is essential for synaptic remodeling during early life, and therefore may have far-reaching behavioral effects.

## 4 Neonatal cross-modal sensory experience

While multisensory integration via direct thalamocortical and intracortical connections across modalities in adults is well appreciated (review: Driver and Noesselt, 2008), less is known about when multisensory connectivity is established, and whether it contributes to the maturation of and the early activity in primary sensory cortices. In developing Mongolian gerbils, several thalamic nuclei project to two or more sensory cortices between P1-P9, and the non-matched modality projections are pruned away after P15 (Henschke et al., 2018). Direct connections between primary visual, auditory, and somatosensory cortices occur later than multisensory interconnections at the thalamic level, occurring at the onset of sensory experience around P15. In addition, the number of multisensory connections drastically increases following the loss of early sensory experience (Henschke et al., 2018), suggesting that sensory experience from other modalities is able to alter the development and function of the somatosensory cortex during early postanal development. In support of this notion, whisker deprivation or dark rearing reduces excitatory synaptic transmission in the correspondent sensory cortex and cross-modally in other sensory cortices through an oxytocin-dependent mechanism (Zheng et al., 2014).

Direct intracortical excitatory connections have also been demonstrated to trigger early synchronous activity in the somatosensory cortex. In mouse slice culture between embryonic day (E)18-P12, waves of spontaneous electrical activity initiate in the septum and ventral cortex and propagate dorsally across the cortex, including the somatosensory region (Conhaim et al., 2011). This is consistent with the recent finding that there is direct, transient excitatory connectivity from the olfactory cortex to the somatosensory cortex, and *in vivo* odor-driven activity propagates broadly across the cortex during the first postnatal week (Cai et al., 2024). This odor-evoked activity enhances whisker-evoked activity in the barrel cortex, while neonatal odor deprivation leads to somatosensory defects in adult mice, suggesting that there is a cross-modal critical window for nasal chemosensation-dependent somatosensory function maturation (Cai et al., 2024). Interestingly, recent work quantifying prenatally active neurons in mice using Targeted Recombination in Active Populations (TRAP) identified the piriform cortex as the most abundantly TRAPed region, indicating that early piriform neurons may represent an interconnected hub-like population whose activity promotes recurrent connectivity in the developing cortex (Wang et al., 2024). In summary, early cross-modal sensory experience, in particular olfactory inputs, can have large impact on the proper maturation and function of the somatosensory cortex.

## 5 Social isolation

During early postnatal life, social isolation is a well-documented stressor that can alter cortical synaptic function. Social isolation in pups has been shown to affect synaptic spine density in the barrel cortex, though the effects vary with different isolation protocols (Bock et al., 2005; Takatsuru et al., 2009). When neonatal rodents are isolated for 6 hours daily during 3-day-long periods in the first and second postnatal week, AMPA receptor trafficking in L4-to-L2/3 synapses in the barrel cortex is significantly reduced in isolated pups when compared to pups that are allowed to remain with littermates (Miyazaki et al., 2012; Miyazaki et al., 2013). These synaptic effects are at least in part attributed to the instability of synapses – increased mushroom spine turnover has been observed as a result of social isolation in pups (Takatsuru et al., 2009; Takatsuru et al., 2015). On the other hand, 3-hour daily isolation from P2 to P14 has been shown to increase neuronal activity in the adult barrel cortex and raise baseline glutamate levels, which, in a pattern not observed in control animals, rises even further following exposure to physical stressors (Toya et al., 2014). These deficits persist after limiting peripheral inputs, which suggests that mechanisms distinct from sensory experience are responsible for the circuit changes associated with social isolation.

Social isolation activates glucocorticoid signaling widely across the brain (Mccormick et al., 1998; review: Cacioppo et al., 2015). Rodents that underwent neonatal isolation show long-term increases in basal corticosterone levels in the barrel cortex (Toya et al., 2014). Elevated glucocorticoid levels have been shown to increase glutamate release in frontal cortical areas (Treccani et al., 2014). Although similar mechanisms have not been directly demonstrated in the barrel cortex, high glucocorticoid levels may also contribute to aberrant glutamatergic homeostasis observed in this region. Indeed, repeated brief exposure to maternal separation results in significantly enhanced spine density in L2/3 pyramidal neurons in the somatosensory cortex (Bock et al., 2005). Acute administration of corticosterone induces higher spine turnover rate in the juvenile barrel cortex compared to control juvenile animals and the turnover rate observed in glucocorticoid treated adults (Liston and Gan, 2011). Pharmacologically decreasing endogenous glucocorticoid signaling through repeated dexamethasone injections decreases spine turnover, and chronic steroid administration eliminates dendritic spines formed prior, in earlier developmental periods (Liston and Gan, 2011). These findings suggest that stress hormones, released in response to a wide variety of stressors including social isolation, can highjack mechanisms of synaptic plasticity during development and have long-term social and cognitive behavioral consequences (Moriceau et al., 2009; review: Sullivan and Holman, 2010).

Recent evidence has identified glial regulation of synaptic function as another potential mechanism through which early social isolation can affect sensory circuit development. Early sensory experience alters cortical synaptic pruning, a process mediated by microglia and astrocytes (Kalambogias et al., 2020; Gesuita et al., 2022). Neonatal social isolation leads to increased microglial process motility both at baseline and in response to sensory stimulation (Takatsuru et al., 2015). Additionally, astrocytes contribute to the developmental changes in L4-to-L2/3 synaptic plasticity by regulating the switch from long-term depression (LTD) to long-term potentiation (LTP) (Martinez-Gallego et al., 2022). The precise mechanistic connections between early life stress, glucocorticoid signaling, microglial and/or astrocytic dysfunction, and long-term synaptic instability are still unclear. Further investigations of glial development in the barrel cortex will be informative for understanding experience-dependent maturation of somatosensory circuits.

## 6 Early life exposure to psychoactive substances

Perinatal exposure to psychoactive substances has also been shown to alter barrel cortex development. The most studied of these substances is ethanol. In humans, consumption of alcohol during pregnancy can cause Fetal Alcohol Syndrome (FAS), a developmental disorder characterized by intellectual disability, facial anomalies, and behavioral deficits (review: Popova et al., 2023). Preclinical rodent models of FAS show that both prenatal and early postnatal (P4-P10) alcohol exposure decrease the area size of the barrel cortex (Miller and Potempa, 1990; Margret et al., 2005, 2006; Powrozek and Zhou, 2005; Chappell et al., 2007). Prenatal ethanol exposure also decreases the number of glia and neurons in the barrel cortex (Miller and Potempa, 1990; Powrozek and Zhou, 2005). This is likely due to the broad apoptotic effect of alcohol exposure in the neonatal brain (Smiley et al., 2019). Between P4 and P7, when alcohol-induced apoptosis reaches maximum levels, intraperitoneal administration of alcohol suppresses spontaneous activity in the barrel cortex and decreases body movements (Lebedeva et al., 2017). Perinatal alcohol exposure decreases the excitability of L5 pyramidal neurons and the cell density of PV interneurons (Granato et al., 2012; Saito et al., 2019). On a behavioral level, adolescent mice exposed to alcohol prenatally show diminished tactile sensitivity (Delatour et al., 2019). These data suggest that early ethanol exposure disrupts normal barrel cortex development and sensory perception by inhibiting cortical activity and increasing apoptosis, which could lead to impaired circuit maturation and organization.

The impact of other psychoactive substances on somatosensory circuit development is less characterized. Prenatal and postnatal opioid exposure has also been shown to increase apoptosis in cortical neurons and microglia, and methadone may specifically decrease the number of GABAergic synapses (Bajic et al., 2013; Grecco et al., 2022). Postnatal (P2-P7) injection of Δ(9)-tetrahydrocannabinol (THC), the primary psychoactive component of cannabis, results in premature retraction and pruning of TCAs during the first postnatal week (Itami et al., 2016). Nicotine, at concentrations similar to those produced by maternal tobacco smoking, desensitizes nicotinic acetylcholine (ACh) receptors in subplate neurons thus diminishing the ACh-driven spontaneous activity in the barrel cortex during the first postnatal week (Hanganu and Luhmann, 2004; Dupont et al., 2006). Overall, these substances may exert long-term effects through pathways that converge upon the disruption of early cortical activity required for sensory circuit development.

## 7 Conclusion

Various types of early life experience—including but not limited to sensory inputs, sleep, social interactions, and substance exposure—exert effects on postnatal circuit organization and contribute to the development of normal or dysfunctional somatosensation. The physiological and environmental factors discussed here will contribute to the understanding of the etiology of atypical sensory perception in many neurodevelopmental and neuropsychiatric disorders, providing considerations on the timing of therapeutic interventions.

## Author contributions

IN: Writing−review and editing, Writing−original draft. AC: Writing−review and editing, Writing−original draft.
